# Next wave of innovation in Alzheimer’s disease therapeutics: The value of novel active transport mechanisms

**DOI:** 10.1002/alz70859_104284

**Published:** 2025-12-25

**Authors:** Catherine J. Mummery, Roberto Villaseñor, Jens Niewoehner, Scarlett Barker, Luka Kulic

**Affiliations:** ^1^ National Hospital for Neurology and Neurosurgery, University College London, London, London United Kingdom; ^2^ F. Hoffmann‐La Roche Ltd, Basel Switzerland; ^3^ Roche Diagnostics GmbH, Penzberg Germany; ^4^ Denali Therapeutics, South San Francisco, CA USA; ^5^ F. Hoffmann‐La Roche Ltd, Basel, Basel Switzerland

## Abstract

**Background:**

The positive study results and approvals for first‐generation anti‐amyloid‐beta (Aβ)‐targeting therapies are an important advancement in the treatment of Alzheimer’s disease (AD), offering new hope for people living with AD and important learnings for the field. We know from existing anti‐amyloid therapy data that rapid and efficient removal of amyloid is needed to achieve significant clinical effects. In addition, the range of targets and modes of action of biologics being explored in AD is increasing. However, delivering large molecules across the blood–brain barrier (BBB) is challenging. The objective of this presentation is to provide an overview of emerging biologic therapeutics and innovative drug delivery methods to actively transport drugs across the BBB.

**Method:**

We will focus on active transport mechanisms in development, which consist of an antibody fragment that binds to the transferrin receptor and harnesses a process called receptor‐mediated transcytosis.

**Result:**

These novel active transport mechanisms have been applied to different modalities, including monoclonal antibodies and antisense oligonucleotides, and have shown clear improvements in the distribution of drugs in preclinical models, such as the Brainshuttle™ and TransportVehicle™ technology described here.

In humans, trontinemab is the first amyloid‐directed Brainshuttle™ antibody fusion to show proof of concept in people living with AD; demonstrating increased brain penetration and amyloid plaque removal with improved safety to date. Latest results from the Phase Ib/IIa Brainshuttle™^M^ AD study in people with mild cognitive impairment due to AD or mild‐to‐moderate AD (NCT04639050) will be presented at this conference.

**Conclusion:**

Utilizing active transport mechanisms, such as the Brainshuttle™ technology, to achieve rapid and efficient amyloid clearance may unlock the full potential of disease modification with Aβ‐targeting monoclonal antibodies in AD. Active transport mechanisms could also be used in other large molecule delivery systems to improve distribution of drugs as well as efficacy and safety profiles.

